# Supplementary material for: Content-rich biological network constructed by mining PubMed abstracts
Source: BMC Bioinformatics. 2004 Oct 8;5:147. doi: 10.1186/1471-2105-5-147 (PMC528731; doi:10.1186/1471-2105-5-147)
Supplement: Additional File 2 — The original results of the above study (non-essential files are deleted to keep the file size under the limit set by BMC bioinformatics). [file 1471-2105-5-147-S2.bz2 › chilibotAdditionalFile2/dip05/410ID11263668E10071/html/BMP2_BMPR1A.html]

 


 **BMP2** and **BMPR1A** 
  
Found 16 abstracts in PubMed, retrieved 05.  
 

 What does Google say? 
 PDF only 
| .edu only 

---

**Interactive relationship** (e.g. stimulation, inhibition, etc)

**Inhibitory relationship**- Using the complex structure of BMP 2  [ **BMP2** ]  bound to its type I receptor ALK3  [ **BMPR1A** ]  as a guide, we introduced extracellular domain ECD mutations in the context of the truncated ALK4 ALK4 trunc construct and assessed the ability of the mutants to inhibit activin function.  Ref: 12665502 J Biol Chem< MedlineTA>J Biol Chem,
**Neutral relationship**

**Non-interactive relationship** (e.g. studied together, co-existance, homology, etc.)

- Our results indicate that there is only a partial overlap of the binding sites on ALK4 and ALK3  [ **BMPR1A** ]  for activin A and BMP 2  [ **BMP2** ] , respectively.  Ref: 12665502 J Biol Chem< MedlineTA>J Biol Chem,
